# Changes in cell wall composition due to a pectin biosynthesis enzyme GAUT10 impact root growth

**DOI:** 10.1093/plphys/kiad465

**Published:** 2023-08-22

**Authors:** Linkan Dash, Sivakumar Swaminathan, Jan Šimura, Caitlin Leigh P Gonzales, Christian Montes, Neel Solanki, Ludvin Mejia, Karin Ljung, Olga A Zabotina, Dior R Kelley

**Affiliations:** Department of Genetics, Development and Cell Biology, Iowa State University, Iowa City, IA 50011, USA; Roy J Carver Department of Biochemistry, Biophysics and Molecular Biology, Iowa State University, Iowa City, IA 50011, USA; Department of Forest Genetics and Plant Physiology, Umeå Plant Science Centre, Swedish University of Agricultural Sciences, Umeå 901 83, Sweden; Department of Genetics, Development and Cell Biology, Iowa State University, Iowa City, IA 50011, USA; Department of Plant Pathology, Entomology, and Microbiology, Iowa State University, Iowa City, IA 50011, USA; Department of Genetics, Development and Cell Biology, Iowa State University, Iowa City, IA 50011, USA; Department of Genetics, Development and Cell Biology, Iowa State University, Iowa City, IA 50011, USA; Department of Forest Genetics and Plant Physiology, Umeå Plant Science Centre, Swedish University of Agricultural Sciences, Umeå 901 83, Sweden; Roy J Carver Department of Biochemistry, Biophysics and Molecular Biology, Iowa State University, Iowa City, IA 50011, USA; Department of Genetics, Development and Cell Biology, Iowa State University, Iowa City, IA 50011, USA

## Abstract

Arabidopsis (*Arabidopsis thaliana*) root development is regulated by multiple dynamic growth cues that require central metabolism pathways such as β-oxidation and auxin. Loss of the pectin biosynthesizing enzyme GALACTURONOSYLTRANSFERASE 10 (GAUT10) leads to a short-root phenotype under sucrose-limited conditions. The present study focused on determining the specific contributions of GAUT10 to pectin composition in primary roots and the underlying defects associated with *gaut10* roots. Using live-cell microscopy, we determined reduced root growth in *gaut10* is due to a reduction in both root apical meristem size and epidermal cell elongation. In addition, GAUT10 was required for normal pectin and hemicellulose composition in primary Arabidopsis roots. Specifically, loss of *GAUT10* led to a reduction in galacturonic acid and xylose in root cell walls and altered the presence of rhamnogalacturonan-I (RG-I) and homogalacturonan (HG) polymers in the root. Transcriptomic analysis of *gaut10* roots compared to wild type uncovered hundreds of genes differentially expressed in the mutant, including genes related to auxin metabolism and peroxisome function. Consistent with these results, both auxin signaling and metabolism were modified in *gaut10* roots. The sucrose-dependent short-root phenotype in *gaut10* was linked to β-oxidation based on hypersensitivity to indole-3-butyric acid (IBA) and an epistatic interaction with *TRANSPORTER OF IBA1* (*TOB1*). Altogether, these data support a growing body of evidence suggesting that pectin composition may influence auxin pathways and peroxisome activity.

## Teaser

During root development, changes in cell wall composition facilitate cell growth properties and auxin metabolism.

## Introduction

Roots are a key organ in plants for water and nutrient acquisition. In angiosperms, the primary root is established during embryogenesis and further elaborated via the coordination of complex cellular processes, including cell wall remodeling and hormone pathways. The plant cell wall is a dynamic structure during root development and can exhibit distinct properties based on cellular function (Somssich et al. 2016). In addition, the plant cell wall provides physical strength to the plant cells and can protect them from internal factors like turgor pressure and external factors like pathogens (Ridley et al. 2001). Because plant cells are held in place through cell walls, the root apical meristem (RAM) must monitor the state of the cell wall to properly coordinate cell divisions (Serra and Robinson 2020; Gu and Rasmussen 2022). While the transcription factor networks regulating the root stem cell populations have been established over time with extensive genetic studies (Sozzani and Iyer-Pascuzzi 2014; Fisher and Sozzani 2016), it is essential to understand the mechanisms beyond transcriptional regulation. In the root, the transition of daughter cells through differentiation leading to their maturation involves regulated cell elongation. Currently, limited information is available regarding cell wall properties within the Arabidopsis (*Arabidopsis thaliana*) RAM (Somssich et al. 2016).

In most angiosperms, including eudicots and nongraminaceous monocots, ∼35% of the primary cell wall is comprised of pectin (Mohnen 2008). Pectins are a family of polysaccharides, of which ∼70% are covalently linked units of galacturonic acid (GalA) residues (Keegstra et al. 1973). Pectins are synthesized in the Golgi apparatus and subsequently transported to the cell wall (Mohnen 2008), where they regulate cell wall properties such as extensibility and thickness (Majda and Robert 2018). The 3 major classes of pectins that are extensively studied are homogalacturonan (HG), rhamnogalacturonan-I (RG-I), and substituted galacturonans that are further subclassified as rhamnogalacturonan-II (RG-II), xylogalacturonan, and apiogalacturonan (Scheller et al. 2007; Mohnen 2008; Lampugnani et al. 2018).

Currently, limited information is available on the cell wall properties of root meristem cells compared to other tissues (Jobert et al. 2023). For example, the differences in the cell wall composition of mitotically dormant QC cells, surrounding stem initials, and the differentiating cell layers are not well understood (Somssich et al. 2016). In addition, the timing and regulation of polysaccharide deposition in differentiating root cells are poorly understood, but a few emergent properties have been described to date (Sinclair et al. 2022). Differentiating plant cells have a thin primary cell wall that facilitates frequent cell division (Baluska et al. 1996). In such dividing cells, callose predominantly constitutes the early stages of the cell plate assembly (Miart et al. 2014; Drakakaki 2015). In addition, pectins and hemicellulose contribute to the cell wall structure by forming a middle lamella that supports intercellular junctions (Drakakaki 2015). Cells at the transition zone within the primary root have a unique cell wall pectin composition, with an abundance of (1→4)-β-d-galactan that demarks the transition from mitotic activity to cell elongation (McCartney et al. 2003).

Galacturonosyltransferases (GAUTs) are a conserved family of enzymes involved in pectin biosynthesis and belong to the glycosyl transferase 8 family (GT8) in the Carbohydrate-Active Enzymes (CAZy) database (Cantarel et al. 2009). Genetic and biochemical characterization of *gaut* mutants revealed their unique role in modulating pectin and xylan polysaccharide composition in shoot tissues and contributing to shoot phenotypes (Caffall et al. 2009; Wang et al. 2013; Lund et al. 2020; Guo et al. 2021; Engle et al. 2022). Fifteen *GAUT* genes annotated in Arabidopsis are phylogenetically classified into 7 clades and 10 *GAUT-like* (*GATL*) genes (Caffall and Mohnen 2009). Two *GAUT* members from clade B-2 have been linked to root growth and development, *GAUT10* and *GAUT15*. *GAUT15* is transcriptionally regulated by auxin and is required for root gravitropism (Lewis et al. 2013). GAUT10 is regulated by auxin posttranscriptionally and is required for primary root growth and lateral root formation (Pu et al. 2019). Loss of *GAUT10* leads to short roots with reduced RAM size in the absence of exogenous sucrose (Pu et al. 2019). In addition, GAUT10 has been shown to be localized to the Golgi and is involved in pectin biosynthesis in Arabidopsis inflorescence and stem tissues (Caffall et al. 2009; Voiniciuc et al. 2018; Guo et al. 2021). GAUT10 is also important for stomata formation in combination with GAUT11, an orthologous gene (Guo et al. 2021). Altogether, these studies suggest that GAUT10 may play numerous roles in plant development.

In order to understand better the biological processes that underpin the short-root phenotype of *gaut10* seedlings, we performed molecular and genetic analyses. This work focused on the *gaut10-3* allele as it has been previously characterized by several research groups as a null allele of *GAUT10* (Caffall et al. 2009; Voiniciuc et al. 2018; Pu et al. 2019; Guo et al. 2021). To facilitate this study, we established a permissive low sucrose growth medium that enabled sufficient growth of *gaut10-3* roots for molecular analyses. Under 0.5% (15 mm) sucrose, the short-root phenotype of *gaut10-3* is due to both a reduction in RAM cell number and impaired epidermal cell elongation. Furthermore, the absence of *GAUT10* impacts auxin-dependent gene expression and metabolism, suggesting that the cell wall composition may influence auxin pathways indirectly. In addition, we have characterized how GAUT10 specifically contributes to root pectin and hemicellulose composition. Genetic analysis with GAUT10 and TRANSPORTER OF IBA1 (TOB1) and indole-3-butyric acid (IBA) response assays indicated that the sucrose-dependent phenotype of *gaut10-3* may be due to altered peroxisome biology. This study provides a working model for GAUT10 in root development and expands our understanding of how cell wall properties can influence root growth.

## Results

### GAUT10 is required for root cell division and elongation

**Figure 1.**
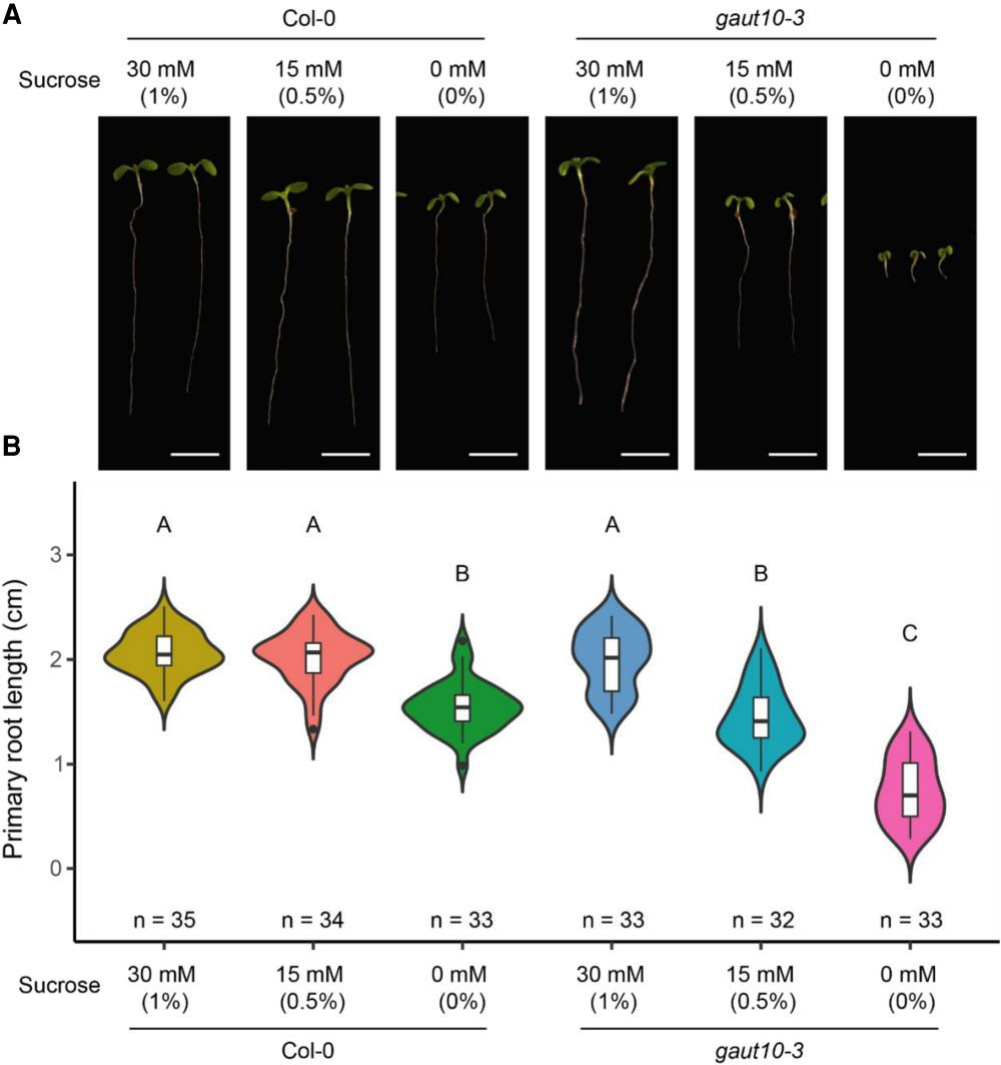
Primary root length is shorter in *gaut10-3* compared to Col-0 grown in 0 and 15 mm sucrose; supplementing the growth medium with 30 mm sucrose rescues the root length phenotype. **A)** Five-day-old *gaut10-3* and Col-0 whole seedlings grown on 0.5× MS supplemented with 3 different sucrose concentrations: 0, 15, and 30 mm. Images were digitally extracted for comparison with all scale bars = 0.5 cm. **B)** Violin plots of quantified root phenotypes in Col-0 and *gaut10-*3. Boxplots within the violin shapes represent the 5 number summaries, where the center line is the median, box limits are the upper and lower quartiles, whiskers are 1.5× interquartile range, and points are outliers. Statistical analysis was performed using a 1-way ANOVA followed by Tukey's post hoc analysis with a *P* < 0.05 to assign letters (A, B, and C) to each genotype/treatment that indicates statistical significance. “*n*” represents the number of biological replicates quantified. Sucrose concentrations are displayed as both a percentage (weight/volume) and molarity (mm).

Through quantitative proteomics coupled with a reverse genetic screen, we previously identified GAUT10 as an auxin downregulated protein required for root development (Clark et al. 2019; Pu et al. 2019). Loss of function alleles of *gaut10* exhibit a short RAM phenotype compared to wild-type Col-0 when grown in 0.5× MS medium lacking sucrose (Pu et al. 2019), and the *gaut10-3* null allele has been well characterized (Caffall et al. 2009; Pu et al. 2019; Guo et al. 2021). Supplementing the 0.5× MS medium with 1% sucrose rescues the *gaut10-3* RAM to wild-type size (Fig. 1). To determine permissive root growth conditions for analyzing *gaut10-3* roots, we grew Col-0 and *gaut10-3* seedlings on 0.5× MS medium supplemented with 0, 0.5%, and 1% sucrose (Fig. 1). Based on this assay, we determined that 5-d-old *gaut10-3* roots are statistically shorter than Col-0 when grown on 0.5× MS supplemented with 0.5% (15 mm) sucrose but still long enough to facilitate molecular and biochemical analyses.

**Figure 2.**
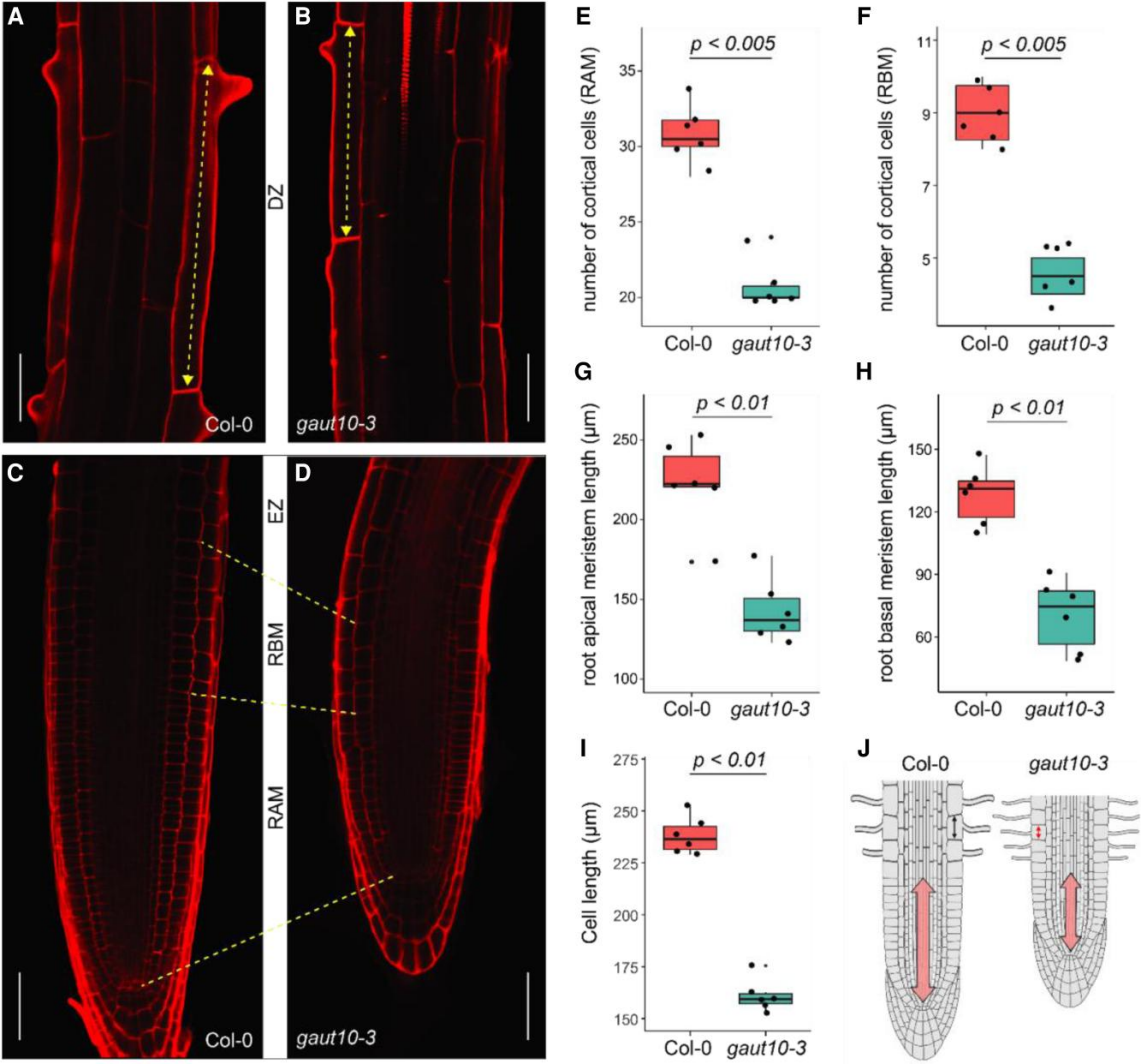
The short phenotype of *gaut10-3* roots is due to a smaller RAM and reduced cell elongation. Images were digitally extracted for comparison. The confocal images (20× magnification) in **A, B)** and **C, D)** show the root DZ and the root MZ of 5-d-old *gaut10-3* and Col-0 seedlings, respectively, with all scale bars showing 20 *μ*m length; the dotted double head arrows in **A, B)** represent the difference in the length of earliest trichoblast cells at the root DZ, and the dotted lines in **C, D)** demark the length of the root apical and basal meristems in *gaut10-3* and Col-0 representative figures. Images are digitally extracted for comparison with all scale bars = 20 *μ*m. Phenotypic differences observed in *gaut10-3* as compared to Col-0 are quantified as box and whisker plots overlayed with scatter plots showing differences in the lengths **G, H)** and the number of cortical cells **E, F)** spanning the apical and the RBM. The meristem lengths are measured on a micrometer (*μ*m) scale. For all phenotypic quantifications pertaining to root meristem **E to I)**, 6 individual seedlings (biological replicates) from each genotype (Col-0 and *gaut10-3*) were used. In **I)**, the average cell lengths of 6 to 10 early differentiating trichoblast cells (technical replicates) from 6 independent roots were quantified. Boxplots **E to I)** represent the 5 number summaries, where the center line is the median, box limits are the upper and lower quartiles, whiskers are 1.5× interquartile range, and points are outliers. The calculated *P*-values **E to I)** are from a nonparametric Wilcoxon rank-sum test used to test the statistical significance of phenotypic differences observed between the 2 genotypes. **J)** A cartoon schematic summarizing the short-root phenotype in *gaut10* roots compared to Col-0, which is due to a smaller RAM (double-ended arrow) and shorter epidermal cells (arrows in the epidermal cells). RAM, root apical meristem; RBM, root basal meristem; MZ, meristematic zone; DZ, differentiation zone.

To determine if the short-root phenotype of *gaut10-3* roots was due to a reduction in cell numbers or cell elongation, we measured these properties in 5-d-old seedlings grown under 0.5% sucrose (Supplemental Table S1). The epidermal cells of *gaut10-3* roots were shorter than Col-0 (Fig. 2, A, B, and I), suggesting that a defect in cell elongation may contribute to the observed short-root phenotype. In addition, we examined the RAM properties of *gaut10-3* grown under 0.5% sucrose in more detail than what had been previously characterized (Pu et al. 2019). The Arabidopsis RAM has been described as consisting of “apical” and “basal” zones (Ishikawa and Evans 1993; Beemster et al. 2003; Verbelen et al. 2006; Zhang et al. 2010; Hacham et al. 2011). The apical region consists of dividing cells of uniform size, while the basal meristem, also called the “transition zone,” is comprised of slow-dividing cells that increase in size. To quantify the smaller RAM properties in *gaut10-3* roots, the number of cells in the apical and basal meristem was measured using a previously described method (Hacham et al. 2011). Compared to Col-0, the number of both apical and root basal meristem (RBM) cells was reduced in *gaut10-3* (Fig. 2, C to H). Altogether these data suggest that *gaut10-3* roots are shorter due to both a reduction in cell number and cell elongation (Fig. 2J).

### Epidermal and lateral root cap marker gene expression is diminished in *gaut10-3*

**Figure 3.**
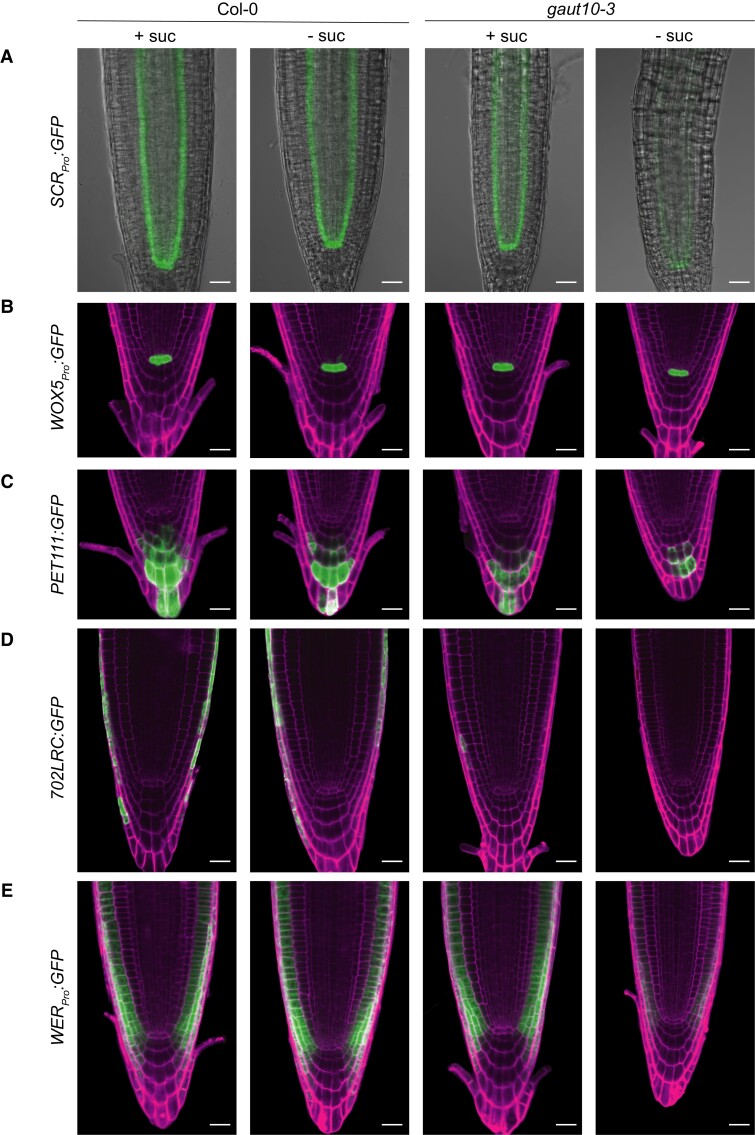
Root cell marker lines are altered in *gaut10-3*. Confocal images of Col-0 and *gaut10-3* 5-d-old primary roots grown in the presence of 15 mm sucrose (+suc) and absence of sucrose (−suc). **A)***SCR:GFP*, which marks the endodermis. **B)***WOX5:GFP*, which marks the quiescent center. **C)***PET111:GFP*, which marks the columella. **D)***702LRC:GFP*, which marks the lateral root cap. **E)***WER:GFP*, which marks the lateral root cap. Scale bars = 20 *µ*m. Images were digitally extracted for comparison.

*GAUT10* mRNA levels have been previously shown to be enriched in several root stem cell types, including the quiescent center, columella, and epidermis/lateral root cap (Epi/LRC) initials (Clark et al. 2019; Supplemental Fig. S1). To determine if the loss of *GAUT10* impacts tissue-specific marker line expression in the RAM, we examined the expression patterns of several well-characterized GFP reporter lines for these cell types. For this experiment, wild-type and *gaut10-3* 5-d-old roots harboring root cell marker lines were grown in the absence (-suc) or presence of 0.5% sucrose (+suc) and imaged via confocal microscopy. In *gaut10-3* roots, the *SCR:GFP* marker, which marks cortex/endodermal cell identity, shows diminished expression in a sucrose-dependent manner (Fig. 3A). In contrast, the QC marker *WOX5:GFP* is expressed normally in *gaut10-3* (Fig. 3B), indicating that the stem cell organizing center is normal. In addition, marker lines for the columella (*PET111:GFP*) and lateral root cap (*702LRC:GFP*) are diminished in *gaut10-3* compared to wild-type independent of sucrose (Fig. 3, C and D). An epidermal marker, *WER:GFP,* showed reduced expression in *gaut10-3* in a sucrose-dependent manner compared to wild type (Fig. 3E). Altogether, these data suggest that *GAUT10* may influence RAM marker gene expression in a sucrose-dependent manner.

### Contributions of GAUT10 to cell wall composition

**Figure 4.**
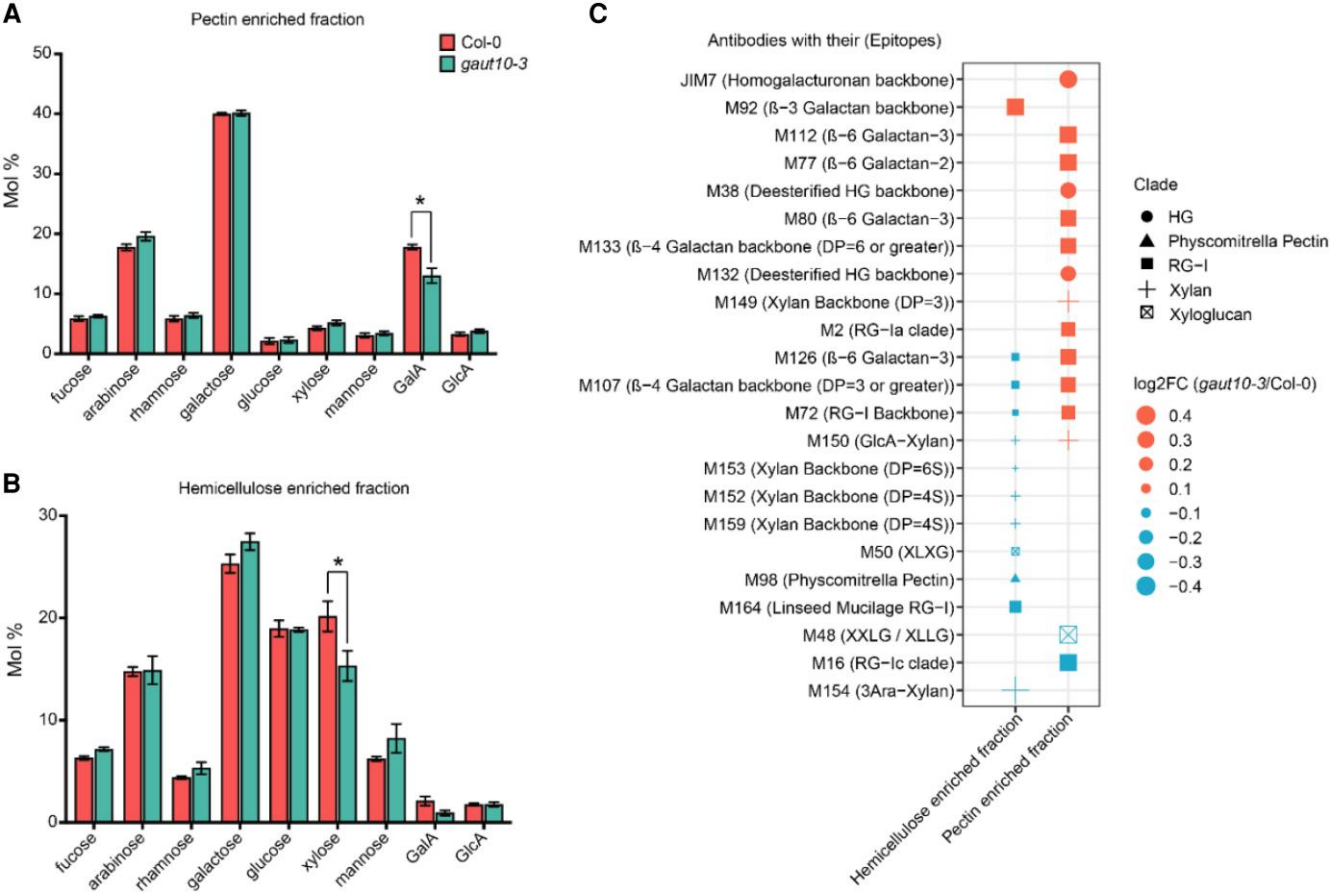
Cell wall polysaccharide composition is altered in *gaut10-3* roots. **A, B)** Concentrations of root cell wall monosaccharides are shown as mole fraction × 100 = mole percentage (Mol%) in the pectin- **A)** and the hemicellulose- **B)** enriched fractions from 3 biological replicates per genotype. The error bars show the Se of the mean (SEM). Statistical analysis was performed using a 2-sample nonparametric Wilcoxon rank-sum test; an asterisk indicates *P* ≤ 0.1. **C)** A 4D dot plot that shows differential binding of cell wall polysaccharide-specific antibodies in root cell wall extracts collected across 3 biological replicates from *gaut10-3* and Col-0 measured by ELISA. The shape annotations show clades of the cell wall–binding antibodies (as defined in Pattathil et al. 2010) whereas the *x* axis indicates the corresponding cell wall fraction origin (i.e. pectin or hemicellulose). The size of the dots represents the log_2_ fold change (*gaut10-3*/Col-0) value calculated using the ELISA absorbance values. Significantly enriched/reduced polysaccharides (23 in total) with their binding epitopes are indicated according to Pattathil et al. (2010) annotations. Statistical significance is determined by a 2-sample nonparametric Wilcoxon rank-sum test with *P* ≤ 0.1. Antibody names and their binding cell wall epitope structures are annotated for each row. HG, homogalacturonan; RG-I, rhamnogalacturonan-I.

GAUT10 has been previously shown to contribute to the pectin biosynthesis of Arabidopsis shoot tissues (Caffall et al. 2009; Guo et al. 2021). To determine how the loss of GAUT10 may impact pectin composition in the root, we performed both monosaccharide composition analysis and glycome profiling (Fig. 4). The monosaccharide analysis of *gaut10-3* roots compared to wild type grown under 0.5% sucrose indicated that GalA levels are reduced in the pectin-enriched fraction of *gaut10-3* roots (Fig. 4A; Supplemental Table S2), which is consistent with a previous report on reduced GalA levels of *gaut10* siliques (Caffall et al. 2009). The total cellulose content in *gaut10-3* roots was the same as Col-0 (Supplemental Table S2). In addition, the xylose levels are significantly reduced in the hemicellulose-enriched fraction of *gaut10-3* roots compared to wild type (Fig. 4B; Supplemental Table S2).

Glycome profiling was performed using a published ELISA-based method on the pectin and the hemicellulose-enriched fractions extracted from 5-d-old Col-0 and *gaut10-3* roots grown under 0.5% sucrose. For this assay, a collection of 66 cell wall–specific antibodies was utilized (Pattathil et al. 2010, 2012). From these analyses, we identified 15 differentially enriched polysaccharide epitopes in the pectin-enriched fraction of *gaut10-3* roots compared to the wild type and 12 in the hemicellulose-enriched fraction (Fig. 4C; Supplemental Table S2).

The epitopes present in the HG backbone (methyl esterified and demethyl esterified), RG-Is (RG-I backbone, RG-Ia, and RG-I/galactans), xylan backbone, and GlcA–xylan were enriched in the CDTA/ammonium oxalate–soluble pectin abundant fraction from *gaut10-3* root cell walls in comparison with the same fraction prepared from Col-0 cell walls (Fig. 4C; Supplemental Table S2). In contrast, RG-Ic and xyloglucan (XXLG/XLLG) are reduced in the pectin-enriched fraction of *gaut10-3* roots compared to Col-0 (Fig. 4C; Supplemental Table S2). In the hemicellulose-enriched fraction, 11 out of 12 significant epitopes were found to be reduced in *gaut10-3* compared to Col-0, including RG-Is (RG-I backbone, RG-I/galactans, and linseed mucilage RG-I), xylans (xylan backbone, GlcA-xylan, 3Ara-xylan), and a xyloglucan (XLXG). Only one significant RG-I epitope, namely the β3-galactan backbone, was found to be enriched in the hemicellulose-enriched cell wall fraction of *gaut10-3* roots in comparison to Col-0 (Fig. 4C; Supplemental Table S2). The observed reduction of xylose in the hemicellulose-enriched fraction (Fig. 4B) corresponds with the reduction of several xylan-related epitopes in glycome profiling of the same fraction (Fig. 4C). Altogether, these data demonstrate that GAUT10 influences RG and XG composition in primary roots.

### Loss of *GAUT10* impacts auxin and cell wall pathway gene expression

**Figure 5.**
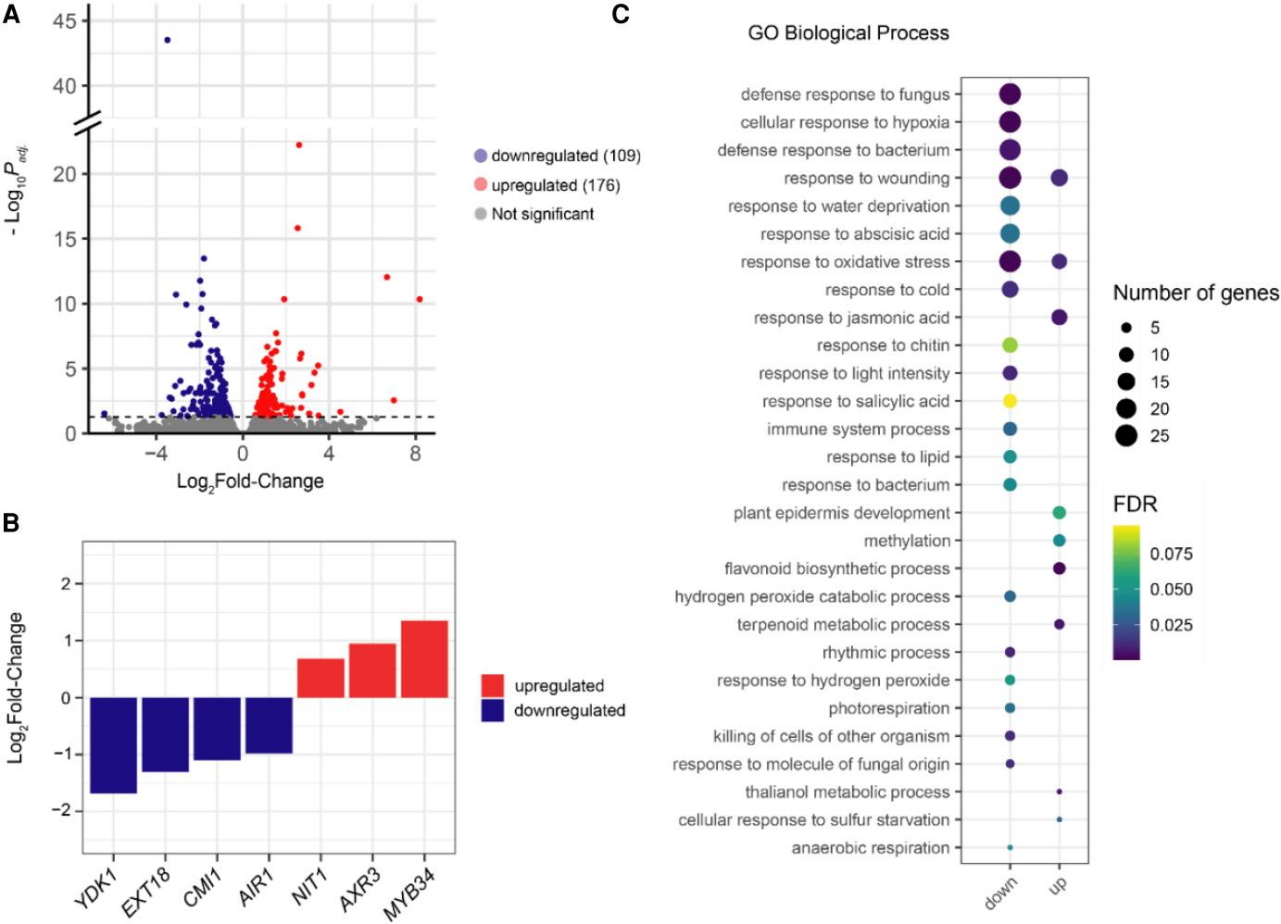
Transcriptomic analysis of 5-d-old *gaut10-3* and Col-0 roots across 3 biological replicates. **A)** Volcano plot showing DEGs with an adjusted *P*_adj._ value (false discovery rate) threshold of 0.05, with log_2_ fold change on the *x* axis and −log_10_(adjusted *P*_adj._ value) on the *y* axis. The upregulated genes (109) and downregulated genes (176) are above the dashed line; transcripts that are not significantly changed are below the dashed line. **B)** Expression values for notable key marker genes are shown in the bar plot with log_2_ fold change on the *y* axis and gene abbreviations on the *x* axis: *YDK1*, *EXT18*, *CMI1*, *AUXIN-INDUCED IN ROOT CULTURES 1* (*AIR1*), *NIT1*, *AXR3*, and *MYB34*. **C)** The top GO terms for biological processes enriched by the DE transcripts in *gaut10-3* compared to Col-0 are shown in a 4D graph, where the size and color of the puncta show the number of DEGs/proteins enriched within each GO term and their statistical significance, respectively.

During root development, there is coordination between cell wall dynamics and gene expression (Taylor-Teeples et al. 2015). To determine how gene expression may be impacted in *gaut10-3* roots, we performed transcriptomics on 5-d-old *gaut10-3* and Col-0 roots grown on 0.5× MS +0.5% sucrose. Differentially expressed genes (DEGs) were identified using the DESeq2 package implemented in R (Love et al. 2014). With an adjusted *P*-value of <0.05, 109 upregulated and 176 downregulated genes were identified in *gaut10-3* roots relative to Col-0 (Fig. 5A; Supplemental Fig. S2 and Table S3). Among these DEGs, we identified several key marker genes that are associated with root development and are consistent with the observed defects in *gaut10-3* roots. For example, *EXTENSIN18* (*EXT18*), which is required for root growth via cell elongation (Choudhary et al. 2015), is downregulated in *gaut10-*3 (Fig. 5B). In addition, several known auxin pathway genes are altered in *gaut10-3* roots including *YADOKARI 1* (*YDK1*), *NITRILASE 1* (*NIT1*), *MYB DOMAIN PROTEIN 34* (*MYB34*), *CA^2+^-DEPENDENT MODULATOR OF ICR1* (*CMI1*), and *INDOLE-3-ACETIC ACID INDUCIBLE 17/AUXIN RESISTANT 3* (*IAA17/AXR3*) (Fig. 5B). Collectively, *YDK1*, *NIT1*, and *MYB34* are important regulators of auxin metabolism (Bartling et al. 1992, 1994; Bartel and Fink 1994; Takase et al. 2004; Celenza et al. 2005; González-Lamothe et al. 2012; Lehmann et al. 2017). *CMI1* is an auxin-regulated gene that modulates auxin responses in the root meristem via a Ca^2+^-dependent pathway (Hazak et al. 2019). Loss of function of *cmi1* results in a shorter primary root with reduced root meristem size (Hazak et al. 2019), which is consistent with the *gaut10-3* root phenotype. *IAA17/AXR3* encodes for an Aux/IAA transcription factor that represses auxin-inducible gene expression (Nagpal et al. 2000; Nakamura et al. 2006; Muto et al. 2007). Altogether, the transcriptomic analysis suggests that the altered cell wall composition in *gaut10-3* may influence auxin pathway gene expression.

In order to identify enriched biological processes among DEGs in *gaut10-3* compared to the wild type, we performed Gene Ontology (GO) enrichment analysis. Overall, numerous GO terms that are typically associated with plant stress and defense are significantly enriched among DEGs in *gaut10-3*. Among upregulated genes, there is an enrichment for “response to hydrogen peroxide” and numerous biotic and abiotic stress terms (Fig. 5C). In addition, 2 classical defense and stress hormone GO terms are enriched (“response to salicylic acid” and “response to abscisic acid”) among the downregulated genes in *gaut10* (Fig. 5C). In contrast, among the upregulated genes in *gaut10-3* are GO biological process (GOBP) terms associated with “plant epidermis development,” “response to jasmonic acid,” and “flavonoid biosynthetic development” (Fig. 5C). This GO analysis suggests that loss of *GAUT10* may impact numerous plant growth and stress responses.

### Local auxin signaling is inhibited in the *gaut10-3* root meristem

**Figure 6.**
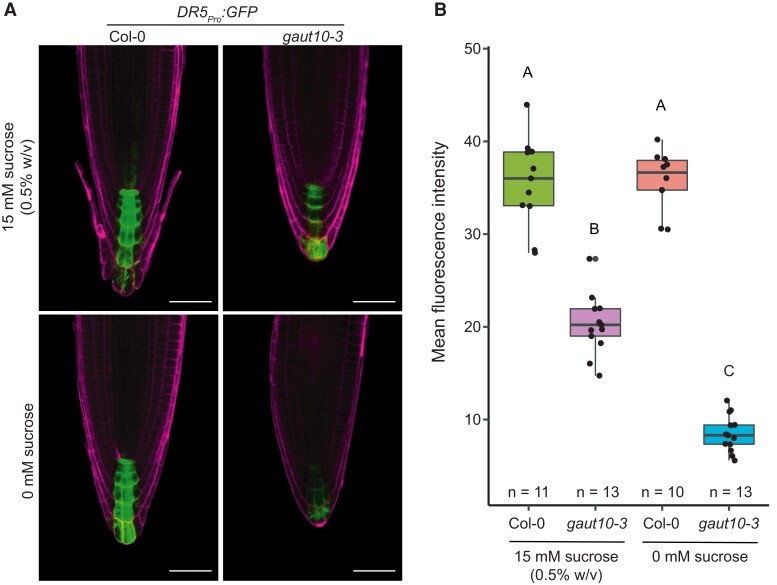
Auxin signaling defects in *gaut10-3* roots compared to wild type. **A)** Representative 20× confocal images of *DR5:GFP* expression in 5-d-old Col-0 and *gaut10-3* roots grown on 0 and 15 mm sucrose. Scale bars = 20 *µ*m. Images were digitally extracted for comparison. **B)** Mean fluorescence intensity (MFI) quantified for each genotype/treatment in *y* axis. Boxplots represent the 5 number summaries, where the center line is the median, box limits are upper and lower quartiles, whiskers are 1.5× interquartile range, and points are outliers. Statistical analysis was a 1-way ANOVA followed by Tukey's post hoc analysis with a *P* < 0.05 to assign letters (A, B, and C) to each genotype/treatment to indicate statistical significance. “*n*” represents the number of biological replicates used for averaging.

The phytohormone auxin regulates much critical growth and developmental cues in plants. Because GAUT10 abundance is regulated by auxin (Pu et al. 2019) and several auxin pathway genes are DE in *gaut10-3* roots (Fig. 5), we wanted to examine auxin signaling using the *DR5:GFP* reporter, which is a synthetic promoter consisting of 7 tandem repeats of an auxin-responsive TCTCTC element and a minimal 35S CaMV promoter driving expression GFP (Friml et al. 2003; Heisler et al. 2005; Hayashi et al. 2014). We observed a significant reduction of *DR5:GFP* expression in *gaut10-3* roots compared to Col-0 (Fig. 6, A and B), which was exacerbated under sucrose-deficit growth conditions (Fig. 6, A and B; Supplemental Table S4). Altogether, these results suggested a defect in local auxin signaling in the *gaut10-3* root meristem.

### Auxin metabolism is altered in *gaut10-3* roots

Auxin homeostasis plays a major role in establishing postembryonic root architecture (Jones and Ljung 2012). In concert with polar auxin transport, local auxin biosynthesis forms the spatiotemporal auxin gradient within the root apex that plays an essential role in root development (Ljung et al. 2005; Petersson et al. 2009; Casanova-Sáez et al. 2021). Plants maintain auxin homeostasis through de novo auxin biosynthesis or by controlling the levels of active auxin through conjugation (primarily amino acids and simple sugars) or by degradation (Normanly 2010; Ruiz Rosquete et al. 2012; Casanova-Sáez et al. 2021). We identified several known auxin metabolite genes as DE in *gaut10-3* roots (Fig. 5B), suggesting that auxin metabolism or signaling may be altered in this mutant. Specifically, *YDK1* conjugates aspartate and other amino acids to IAA, consequently lowering active auxin levels in vivo in a tissue-specific manner (Takase et al. 2004; González-Lamothe et al. 2012). *NIT1* encodes an enzyme that catalyzes the conversion of indole-3-acetonitrile (IAN) to indole-3-acetamide (IAM) as it modulates IAA biosynthesis (Bartling et al. 1992, 1994; Bartel and Fink 1994; Lehmann et al. 2017). *NIT1* overexpression results in shorter primary roots in seedlings, explaining its role in regulating root development via auxin homeostasis (Lehmann et al. 2017). *MYB34* plays a key role in the transcriptional regulation of enzymes that maintain the metabolic homeostasis of tryptophan (TRP) and indole glucosinolates in Arabidopsis (Celenza et al. 2005). To determine how the observed transcriptional changes to auxin metabolism genes in *gaut10-3* roots impact auxin metabolism, we measured auxin metabolites in Col-0 and *gaut10-3* roots grown in the presence of 0.5% sucrose.

**Figure 7.**
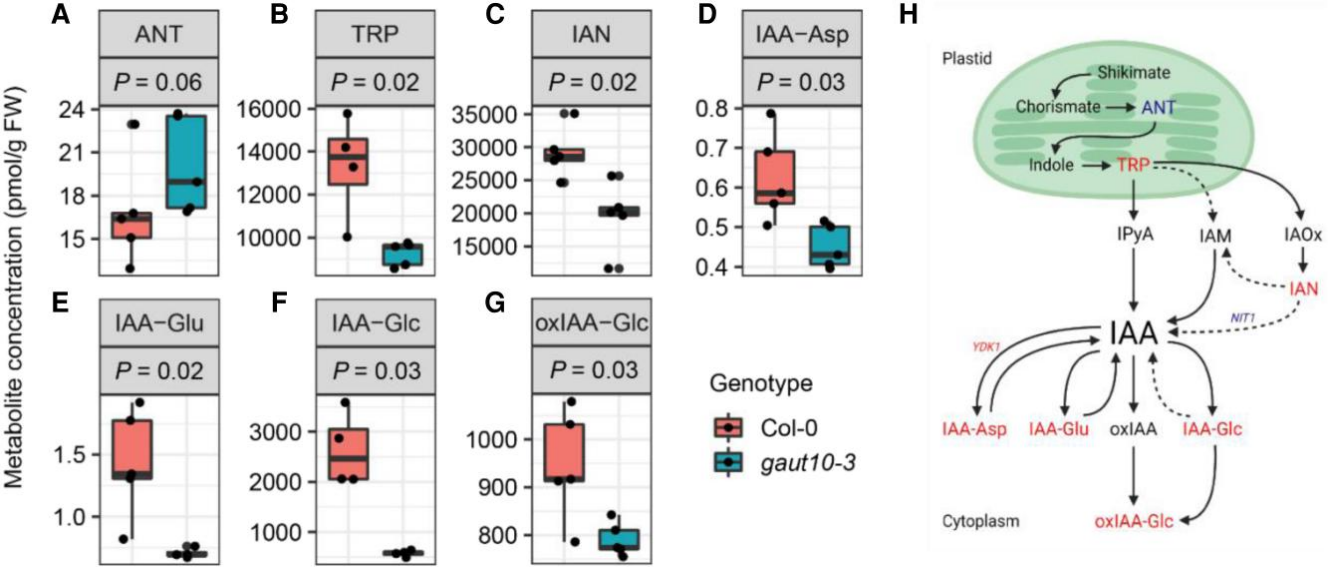
Auxin metabolism is altered in *gaut10-3* roots compared to wild type. Metabolite quantification by LC–MS/MS visualized by box and whisker plots for with the corresponding *P*-value (*P*) indicated for each metabolite across 5 biological replicates per genotype. For all metabolites quantified, a 2-sample nonparametric Wilcoxon rank-sum test followed by Benjamini–Hochberg correction for multiple testing was performed to identify significantly altered metabolites with *P* ≤ 0.1. **A)** ANT. **B)** TRP. **C)** IAN. **D)** IAA–Asp. **E)** IAA–Glu. **F)** IAA–Glc. **G)** oxIAA–Glc. Boxplots **A to G)** represent the 5 number summaries, where the center line is the median, box limits are the upper and lower quartiles, whiskers are 1.5× interquartile range, and the points are outliers. **H)** Summary model figure showing annotated auxin biosynthesis pathways. Reduced metabolites are indicated in *gaut10-3* roots in red text; likewise, pathway enzymes shown in red are downregulated, whereas the genes in blue text show increased transcript levels. The solid arrows indicate pathways that have known enzymes, genes, and intermediates, while dashed arrows indicate pathways that are not well defined.

IAA levels are the same in *gaut10-3* compared to wild type (Supplemental Fig. S3); however, several IAA precursors and conjugates were altered in abundance (Fig. 7). Anthranilate (ANT), the primary rate-limiting precursor of TRP biosynthesis, was increased in *gaut10-3* roots compared to Col-0 (Fig. 7A). In contrast, TRP levels were significantly reduced in *gaut10-3* roots compared to Col-0 (Fig. 7B). IAN, which is debatably involved in indole-3-acetaldoxime (IAOx) to IAA conversion (Sugawara et al. 2009; Casanova-Sáez et al. 2021), was depleted in *gaut10-3* roots (Fig. 7C). In addition, several inactive forms of IAA were significantly decreased in *gaut10-*3 compared to wild type, including IAA–aspartate (IAA–Asp), IAA–glutamate (IAA–Glu), IAA–glucose (IAA–Glc), and 2-oxindole-3-acetic acid–Glc (oxIAA–Glc) (Fig. 7, D to G). The observed reduction of IAN and IAA–Asp and increase in ANT are consistent with the differential expression of their respective metabolic enzymes (*YDK1*, *NIT1*, and *MYB34*) in *gaut10-3* roots (Fig. 7H). Collectively, these data suggest that pectin composition may influence auxin metabolism within primary roots.

### Genetic interactions between GAUT10 and TOB1

**Figure 8.**
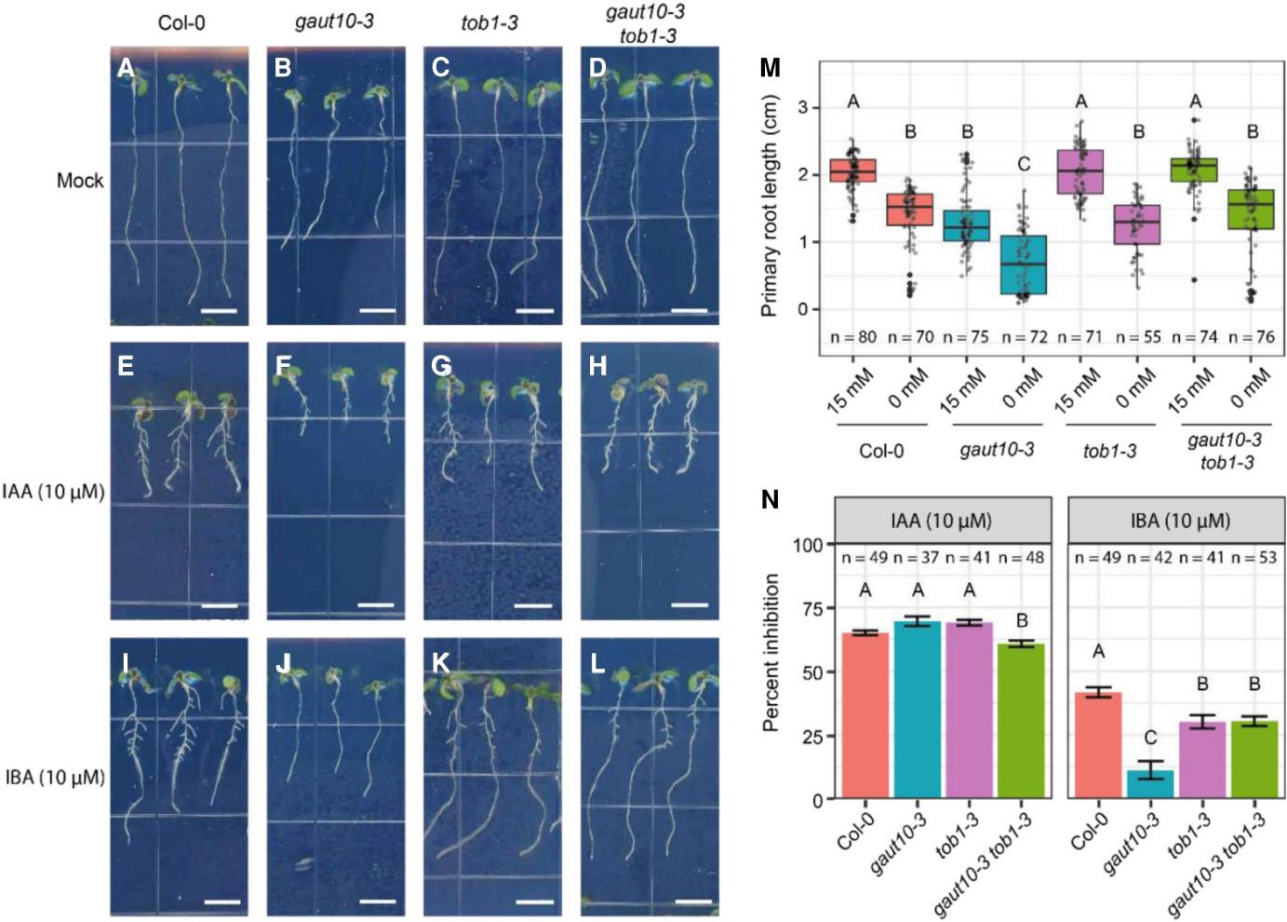
Genetic interaction between *gaut10* and *tob1*. **A, E, I)** Wild-type Col-0 roots grown under 0.5% sucrose (mock), 0.5% sucrose + 10 mm IBA, or 0.5% sucrose + 10 mm IAA. **B, F, J)***gaut10-3* roots grown under 0.5% sucrose (mock), 0.5% sucrose + 10 mm IBA, or 0.5% sucrose + 10 mm IAA. **C, G, K)***tob1-3* roots grown under 0.5% sucrose (mock), 0.5% sucrose + 10 mm IBA, or 0.5% sucrose + 10 mm IAA. **D, H, L)***gaut10-3 tob1-3* roots grown under 0.5% sucrose (mock), 0.5% sucrose + 10 mm IBA, or 0.5% sucrose + 10 mm IAA. Images in **A to L)** were digitally extracted for comparison with all scale bars = 0.5 cm. **M)** Quantification of primary root length of Col-0, *gaut10-3*, *tob1-3*, and *gaut10-3 tob1-3*. Boxplots represent the 5 number summaries, where the center line is the median, box limits are the upper and lower quartiles, whiskers are 1.5× interquartile range, and points are outliers. **N)** IBA and IAA responses in Col-0, *gaut10-3*, *tob1-3*, and *gaut10-3 tob1-3*. All statistical analysis was a 1-way ANOVA followed by Tukey's post hoc analysis with a *P* < 0.1 to assign letters (A, B, and C) to each genotype/treatment to indicate statistical significance. “*n*” represents the number of biological replicates used for averaging in all cases. The error bars represent the Se.

To infer possible mechanisms associated with the sucrose-dependent phenotype of *gaut10-3* roots, we examined the transcriptomic data (Supplemental Table S5). Sucrose-dependent root growth in Arabidopsis has been linked to numerous pathways, including sugar signaling, sugar transport, and lipid metabolism via the peroxisome (Woodward 2005; Marzec and Kurczynska 2014; Urano et al. 2016; Wang et al. 2021). Among the DEGs in *gaut10-3* roots, there were no hallmark sugar-signaling genes. However, 2 genes related to peroxisome function that were altered in *gaut10-3* roots include *TOB1* and *PEROXIN 11C* (Supplemental Table S3). Peroxisome-defective mutants exhibit hallmark sucrose-dependent short-root phenotypes (Strader et al. 2011), suggesting that such dysfunction may underpin the *gaut10-3* phenotype. In *gaut10-3* roots, *TOB1* transcript is elevated while *PEX11C* expression is decreased. To examine potential genetic interactions between GAUT10 and TOB1, we generated a *gaut10-3 tob1-3* double mutant. Previous work established that *gaut10-3* roots respond normally to IAA (Pu et al. 2019), but the response of *gaut10-3* to IBA had not been characterized in this mutant. The response to IBA and IAA was examined in *gaut10-3*, *tob1-3*, and *gaut10-3 tob1-3* roots (Fig. 8). Both wild-type and *gaut10-3* roots are inhibited to exogenous IAA treatment while *tob1-3* roots are slightly insensitive, as previously reported (Michniewicz et al. 2019) (Fig. 8, A, H, and N). Both *tob1-3* and *gaut10-3* are insensitive to IBA, and the *gaut10-3 tob1-3* double mutant exhibits a response like *tob1-3* (Fig. 8N). In addition, the short-root phenotype of *gaut10-*3 is suppressed in the *gaut10-3 tob1-3* background (Fig. 8M). Altogether, these data suggest an epistatic interaction between *GAUT10* and *TOB1*.

## Discussion

In the elongation zone of the primary root, cells transition from the meristematic zone (MZ) and rapidly elongate along the longitudinal axis while preventing any lateral expansion of cells that require cell wall remodeling (Ledbetter and Porter 1963; Schiefelbein and Benfey 1991; Somssich et al. 2016). Cell wall remodeling enzymes largely contribute to such unidimensional cell expansions in response to internal turgor pressure (Ledbetter and Porter 1963; Cosgrove 2005). GalA is one of the main monosaccharides constituting all pectin polysaccharides, including HG, RG-I, RG-II, and xylogalacturonan (Caffall and Mohnen 2009). Many of the characterized *GAUT* mutants display substantial reductions in the amount of GalA present in their cell walls prepared from various above-the-ground parts of the plants (Caffall et al. 2009; Voiniciuc et al. 2018; Guo et al. 2021); however, some mutants like *gaut13* and *gaut14* showed higher content of GalA in their siliques and inflorescences (Caffall et al. 2009). To our knowledge, roots have not been analyzed in any *gaut* mutant plants. Here, we demonstrated that *gaut10-3* roots also have cell walls with reduced GalA and xylose. Our results are consistent with previous results obtained for *gaut10-1* and *gaut10-2* inflorescence and siliques (Caffall et al. 2009). Double *gaut10 gaut11* plants also showed reduced pectin biosynthesis in stomata cells (Guo et al. 2021). Recombinant GAUT10 showed catalytic activity when given UDP–GalA as a substrate; however, the activity was very low, and it has been proposed that GAUT10 may have a unique role in synthesizing either a unique HG compared to other GAUTs (such as GAUT1) or an HG connected to RG-I (Engle et al. 2022).

Monosaccharides such as GalA and xylose are significantly reduced in the pectin and hemicellulose-enriched cell wall fractions of *gaut10-3* roots, respectively, which showed a net decrease in GalA and xylose containing polysaccharides in the *gaut10-3* root cell wall as compared to Col-0. The glycome analysis performed here of both cell wall fractions observed significant alterations in the levels of specific polysaccharides, which is consistent with the monosaccharide analysis. Antibodies specific to GalA containing RG-I and HG epitopes were enriched in the pectin fraction. We also observed a significant decrease in RG-I, xylan, and xyloglucan-related epitopes in the hemicellulose-enriched fraction. The increased HG and multiple RG-I pectin–related epitopes, specifically in pectin fraction, might indicate the higher extractability of polysaccharides carrying such epitopes due to their weaker binding nature in the *gaut10-3* cell wall in comparison with cell walls of Col-0. For example, epitopes recognized by 4 particular cell wall antibodies (M126, M107, M72, and M150) were increased in the more soluble fraction extracted with CDTA–ammonium oxalate but consequently reduced in the following extract with a strong alkali, suggesting the increased solubility of the corresponding polysaccharide molecules within *gaut10* roots. It is also possible that cell wall reinforcement in *gaut10* roots is loosened, resulting in reduced cell expansion and suppressed growth that is necessary to compensate for the changes in how specific polysaccharides are integrated into cell walls. In the future, more detailed studies will be needed to clarify the impact of loss of GAUT10 on the specific polysaccharide structure/interactions and the general impact on cell wall organization in root cells.

**Figure 9.**
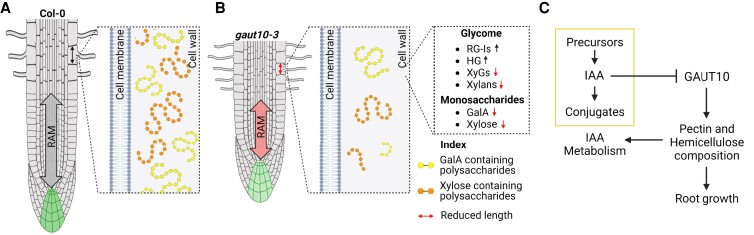
Working model for roles of GAUT10 in root morphogenesis. **A)** In wild-type Col-0 roots, normal cell wall composition leads to correct RAM size and *DR5:GFP* maxima in the QC and columella. **B)** In the absence of GAUT10, pectin composition is altered leading to reduced cell expansion and *DR5:GFP* expression. **C)** A molecular framework for GAUT10 function based on biochemical and genetic analyses of *gaut10-3* roots. IAA represses GAUT10 protein accumulation, which is a positive regulator of pectin and hemicellulose formation in roots. GAUT10-dependent cell wall composition is required for primary root morphogenesis and for auxin metabolism.

Interestingly, *gaut10* siliques and inflorescences did not show a reduction of xylose (Caffall et al. 2009); however, a reduction of both GalA and xylose was observed earlier in another *gaut* mutant, *quasimodo 1* (*qua1/gaut8*) (Orfila et al. 2005). Pectic galactans have been associated with wall strengthening (McCartney et al. 2000), and the higher levels of pectin-specific epitopes in *gaut10-3* mutant roots in comparison with Col-0 roots may also be a result of cell wall structural changes leading to increased cell wall rigidity in mutant root cells resulting in suppression of their growth. Altogether, we can infer that GAUT10 plays a critical role in synthesizing/maintaining pectic polysaccharides within primary root cell walls (Fig. 9).

GAUT10 was identified as an auxin downregulated protein (Clark et al. 2019; Pu et al. 2019) with roles in the regulation of RAM size by a reverse genetic study with 3 loss-of-function alleles (Pu et al. 2019). Here, we performed transcriptomics and auxin metabolomics on *gaut10-3* to substantiate its role as a cell wall enzyme that interacts with auxin pathways and further delineate how GAUT10 function may be linked to auxin biology. Transcriptomic analysis of *gaut10-3* roots implicated auxin metabolism and signaling associated with the short-root phenotype, which was validated by *DR5:GFP* expression analysis and auxin metabolite quantification. In the absence of GAUT10, both IAA precursors and inactive conjugates were altered. Within the lateral root cap, cytokinin promotes the degradation of IAA into IAA–Glu, an inactive conjugate, thereby controlling root meristem size and root growth by inducing cell differentiation (di Mambro et al. 2019). The diminished expression of *702LRC:GFP* in *gaut10-3* roots indicates that the lateral root cap may be altered, although the cell files appear normal, which might explain the observed decrease in IAA–Glu (along with other IAA conjugates). Previous work has shown that modulation of IAA conjugation and GH3 activity can influence root morphogenesis in Arabidopsis (Mateo-Bonmatí et al. 2021; Casanova-Sáez et al. 2022). The observed decrease of numerous IAA conjugates (IAA–Asp, IAA–Glu, IAA–Glc, and oxIAA–Glc) in *gaut10-3* roots suggests that IAA homeostasis is influenced by cell wall properties. The differential expression of enzymes catalyzing the conversion of these auxin conjugates and precursors in *gaut10-3* compared to wild-type roots is consistent with this working model (Fig. 7H).

The short-root phenotype in *gaut10-3* manifests due to both a reduction in RAM size and reduced elongation of mature epidermal cells, suggesting that pectin composition can influence both cell proliferation and elongation during root growth. Indeed, the role of GAUT10 in influencing cell elongation properties is further supported by overexpression of genes enriched for the “plant epidermis development” GO term in the *gaut10-3* roots. Root morphogenesis can be studied by visualizing the expression of cell-type–specific markers (Bargmann et al. 2013). In wild-type Col-0 roots, the expression of several cell-type–specific marker lines was not altered by the presence or absence of sucrose (Fig. 3). This result is in line with a recent report which demonstrated that sucrose can influence the relative proportion of cells within a cell type but overall does not alter cell type identities (Shulse et al. 2019). In contrast, we observed reduced expression of several root cell marker lines (*SCR:GFP*, *WER:GFP*, *PET111:GFP*, and *702LRC:GFP*) in *gaut10-3* in the absence of sucrose while expression of the QC marker *WOX5:GFP* was normal. However, the corresponding radial and longitudinal cell patterning in *gaut10-3* roots appears normal. These results may be interpreted such that marker gene expression is diminished due to a change in pectin composition, but organ patterning remains normal in the absence of GAUT10. Given that numerous plant growth and stress genes are DE in *gaut10-3* roots, these data suggest several downstream cellular processes may be impaired when pectin composition is altered. Future investigations of cell wall composition across the developing primary root may be informative for identifying which cell-type–specific contexts are associated with particular HG or RG moieties.

Sucrose positively regulates root stem cell activation and promotes root epidermal cell elongation in Arabidopsis (Wu et al. 2019; Dong et al. 2022). The sucrose-dependent root growth defects of *gaut10-3* suggest a potential link between cell wall composition and sucrose-dependent growth. Notably, links between cell wall dynamics and central metabolism have been reported in studies on *trehalose-6-phosphate synthase1* (*tps1*) (Gómez et al. 2006), glycosyl hydrolases (Lee et al. 2007), *high sugar response8* (*hsr8*), and *murus* (*mur*) mutants (Li et al. 2007). In the absence of *TPS1*, which is required for the conversion of UDP-Glc to Glc-6P, pectin composition is altered, and the expression of numerous cell wall pathway genes is altered (Gómez et al. 2006). These data suggest that alteration in trehalose metabolism can impact cell wall metabolism. In addition, sugar-hypersensitive phenotypes have been reported for several enzymes that contribute to cell wall formation, namely glycosyl hydrolases and arabinose synthesis mutants, namely β-galactosidase, β-xylosidase, β-glucosidase, *hsr8/mur4*, *mur1*, and *mur3* (Lee et al. 2007; Li et al. 2007). Characterization of *hsr8/mur4* seedlings led to a proposed pathway from the cell wall to the nucleus that influences sugar-mediated growth (Li et al. 2007). More investigations into this observed phenomenon will be required to understand better the signal transduction pathway(s) between the cell wall and central carbon metabolism.

Several complex pathways are known to influence sugar-dependent growth in Arabidopsis including SUCROSE NON-FERMENTING 1 (SNF1)-RELATED KINASE 1 (SnRK1), TARGET OF RAPAMYCIN (TOR), HEXOKINASE 1 (HXK1), trehalose 6-phosphate (Tre6P), and β-oxidation (Rinaldi et al. 2016; Fichtner et al. 2021). It was not known which pathway(s) were impaired in *gaut10-3* roots to explain the observed sugar-dependent short-root phenotype. Based on the transcriptomic analysis of *gaut10-3*, it appeared that alteration of β-oxidation–based processes could explain the sucrose-dependent short-root phenotype, which is a hallmark of this pathway (Rinaldi et al. 2016). Specifically, the expression of TOB1 was upregulated in *gaut10-3*, while *PEX11C* was decreased. In plant cells, the conversion of IBA to IAA requires functional peroxisomes and IBA transport via TOB1 (Damodaran and Strader 2019; Michniewicz et al. 2019). The altered response to IBA but normal response to IAA in *gaut10-3* roots is consistent with altered peroxisome function. In addition, the observed epistatic interaction between *TOB1* and *GAUT10* supports a working model that links the cell wall composition to IAA metabolism (Fig. 9C). Thus, it appears that the short-root phenotype of *gaut10-3* can be rescued by exogenous sucrose due to peroxisome dysfunction. Notably, other studies have also uncovered links between cell wall status and peroxisome function (Kanai et al. 2010; Rinaldi et al. 2016; Dai et al. 2019). Peroxisome defective mutants identified from forward genetic screens include PECTIN MEHTYLESTERASE31 (PME31) and ANTHER DEHISCENCE REPRESSOR (ADR), which both contribute to cell wall composition (Rinaldi et al. 2016; Dai et al. 2019). In addition, alteration of a peroxisomal ABC transporter PED3/PXA1/CTS can impair pectin composition in seeds (Kanai et al. 2010). Collectively, these studies suggest that cell wall composition may feedback to auxin homeostasis via several distinct pathways to influence root morphogenesis.

## Materials and methods

### Plant material

All seed stocks used in this study were obtained from the Arabidopsis Biological Resource Center (ABRC) at Ohio State University. Arabidopsis (*A. thaliana*) plants used in this study were all in the Col-0 background. SALK_092577 corresponds to the *gaut10-3* null allele, which has been previously characterized (Caffall et al. 2009; Voiniciuc et al. 2018; Pu et al. 2019; Guo et al. 2021). SALK_205450 corresponds to the *tob1-3* knockdown allele, previously characterized as a knockdown allele (Michniewicz et al. 2019). The *WEREWOLF:GFP* (epidermal cell marker), *702LRC:GFP* (lateral root cap marker), *SCARECROW:GFP* (cortex/endodermal cell marker), *PET111:GFP* (columella cell marker), and *DR5:GFP* lines have been previously described (Friml et al. 2003; Heisler et al. 2005; Bargmann et al. 2013; Hayashi et al. 2014).

For phenotyping assays, seeds were surface sterilized using 50% bleach (*v*/*v*) and 0.01% Triton X-100 (*v*/*v*) for 10 min and then washed 5 times with sterile water. Seeds were then imbibed in sterile water for 2 d at 4 °C and then transferred to 0.5× MS plates supplemented with 15 mm sucrose and 0.8% agar (*w*/*v*). Seedlings were grown under long-day photoperiods (16 h light/8 h dark) at 23 °C in a Percival growth chamber.

### Root phenotyping

Intact seedlings (5-d-old) were imaged on a flatbed scanner (Epson V600). Measurements of primary root lengths were performed using ImageJ. Roots were stained with propidium iodide (PI) and imaged under a confocal microscope for root meristem and differentiation zone (DZ) phenotyping using a 20× objective. Measurements of root meristem lengths (apical and basal) and the epidermal cells at the DZ were performed using ImageJ, and the number of cortical cells was counted across 6 biological replicates as previously described (Hacham et al. 2011). Individual cells (technical replicates) were measured from 6 independent roots per genotype (biological replicates). Two-sample nonparametric Wilcoxon rank-sum tests were performed to assess statistical significance.

### Genotyping

All primers used for genotyping are provided in Supplemental Table S6 based on previously published studies (Caffall et al. 2009; Michniewicz et al. 2019; Pu et al. 2019).

### Confocal microscopy

Homozygous *gaut10-3* plants were crossed with the following transgenic lines: *WEREWOLF:GFP*, *702LRC:GFP*, *SCARECROW:GFP*, *PET111:GFP*, and *DR5:GFP*. After the initial crosses, F1 seedlings were genotyped by PCR using T-DNA and transgene-specific primers and confirmed to be heterozygous for *GAUT10/gaut10-3* and carrying the correct *GFP* transgenes. F1 plants were selfed and then carried through to the F3 generation; genotypes were verified by PCR at each generation. F3 individuals that were homozygous for *gaut10-3* and each GFP reporter were bulked for imaging. Five-day-old roots of these double transgenic and corresponding control transgenic lines were stained with PI and imaged under a 20× objective on a Zeiss LSM 700 confocal microscope. GFP was imaged with excitation at 488 nm and collection at 555 nm. PI was imaged with excitation at 555 nm and collection at 640 nm. For all experiments, the gain was set to 650, and the intensity was set to 2%. GFP fluorescence intensity was calculated in ImageJ as previously described (Lisi et al. 2012; Kim et al. 2017). In total, 6 to 10 biological replicates were imaged for each genotype under each growth condition.

### Transcriptomic profiling

Transcriptomic analyses were performed as previously described (Dash et al. 2021). Col-0 and homozygous *gaut10-3* seeds were surface sterilized, followed by cold stratification for 3 d in the dark at 4 °C. Next, they were plated on 0.5× MS + 15 mm sucrose plates overlaid with sterile 100-*µ* nylon mesh squares to facilitate tissue harvesting. Seedlings were grown under long-day photoperiods (16 h light/8 h dark) at 23 °C. Roots from 5-d-old seedlings were dissected using surgical knives and then weighed and snap frozen in liquid nitrogen; approximately 100 mg of seedling root tissue was collected per replicate/genotype. Three independent biological replicates were generated for each genotype. Snap-frozen roots were ground to a fine powder in liquid nitrogen using a mortar and pestle. Total RNA was extracted from the root samples using TRIzol, followed by column clean-up using the Quick-RNA plant kit (Zymo research). Total RNA concentration was estimated using a NanoDrop and Qubit. RNA quality was checked via Bioanalyzer at the ISU DNA Facility. QuantSeq 3′ mRNA libraries were prepared using the Lexogen 3′ mRNA-seq FWD kit and sequenced on an Illumina HiSeq 3000 as 50 bp reads at the ISU DNA Facility. QuantSeq reads were mapped to the TAIR10 genome using STAR (Dobin et al. 2013), and differential gene expression analysis was performed using DeSeq2 implemented in R (Love et al. 2014). Transcripts with an false discovery rate (FDR) cutoff of <0.05 and a log_2_ fold change ≥ 0.5 were defined as DE.

### GO analysis

DEGs were tested for statistical overrepresentation of GOBP terms using Panther and the *A. thaliana* database. A standard Fisher’s exact test with FDR correction was used to calculate GO term enrichment against the total set of detected transcripts as the reference. Top GOBP terms were plotted on the *y* axis against their genotype/treatments on the *x* axis in multidimensional dot plots.

### Cell wall, pectin, and hemicellulose extraction

Total cell wall material, pectin, and hemicellulose fractions from 3 biological replications of Col-0 and *gaut10-3* root samples grown in 0.5× MS + 15 mm sucrose growth medium were prepared as previously described (Zabotina et al. 2012). Briefly, the root samples were ground to a fine powder in liquid nitrogen, and the cell wall was extracted by using successive organic solvents, starting with 80% (*v*/*v*) ethanol, followed by 80% (*v*/*v*) acetone, chloroform:methanol (1:1, *v*/*v*), and finally with 100% acetone. The resultant cell wall material was air dried, and the pectin and hemicellulose polysaccharides were extracted successively by using 50 mm CDTA:50 mm ammonium oxalate (1:1) buffer (*v*:*v*) and 4 m KOH, respectively. The extracts were neutralized with acetic acid, dialyzed against water, and dried by lyophilization. The dried pectin-enriched and hemicellulose-enriched extracts were stored at 4 °C for further analysis.

### Glycome profiling of epitopes of pectin and hemicellulose extracts

Glycome profiling was performed as previously described (Pattathil et al. 2010, 2012). The amount of sugar in the pectin and hemicellulose extracts was determined by the phenol–sulfuric acid method, and each ELISA well was loaded with an equal amount of polysaccharide sample dissolved in water (50 *µ*L/well from a 60 ng/*µ*L solution). Glycome profiling was carried out using 66 different mouse primary glycome antibodies purchased from the Complex Carbohydrate Research Center (University of Georgia) as previously described (Pattathil et al. 2010, 2012; Zabotina et al. 2012). The color development was detected at 450 nm wavelength using a plate reader, and each sample’s optical density (OD) reading was statistically analyzed and compared. The ELISA assays were repeated in triplicate for both the pectin and hemicellulose extracts. Statistical analysis was performed using a nonparametric Wilcoxon rank-sum test with a *P* ≤ 0.1 to compare mean OD_450_ absorbances between Col-0 and *gaut10-3*.

### Monosaccharide composition analysis

Monosaccharide composition was determined according to previously described methods (Brenner et al. 2012). One milligram of cell wall was hydrolyzed with 2 m trifluoroacetic acid (TFA) and analyzed by high-performance anion-exchange chromatography with pulsed amperometric detection (HPAEC–PAD) (Dionex, Sunnyvale, CA) using a CarboPac PA20 column. The column was calibrated using monosaccharide standards purchased from Sigma-Aldrich, which included L254fucose (l-Fuc), l-rhamnose (l-Rha), l-arabinose (l-Ara), d-galactose (d-Gal), D255Glc (d-Glc), d-xylose (d-Xyl), d-mannose (d-Man), d-GalA, and d-glucuronic acid (D256GlcA) (Brenner *et al.* 2012). The content of different monosaccharides in the hydrolysates analyzed was estimated as a molecular fraction (mol%). Statistical analysis was performed using a nonparametric Wilcoxon rank-sum test with a *P* ≤ 0.1 to compare mean monosaccharide concentrations between Col-0 and *gaut10-3*.

### Cellulose content measurements

The cellulose content present in the cell wall of Col-0 and *gaut10-3* roots was estimated by a modified Updegraff reagent method (Updegraff 1969; Kumar and Turner 2015). From each sample, 75 to 80 mg of cell wall was placed in a preweighed screw cap glass tube, and 2 mL of Updegraff reagent (acetic acid:nitric acid:water, 8:1:2 *v*/*v*) was added. The glass tubes were tightly closed with polytetrafluoroethylene (PTFE) seal caps. Subsequently, the tubes were incubated in a boiling water bath for 30 min, cooled to room temperature, and centrifuged at 1750 × *g* rpm for 10 min to pellet down the cellulose. The supernatant was removed by aspiration. The cellulose pellet was washed 5 times with water and twice with 100% acetone by repeated centrifugation and aspiration. The resultant cellulose was dried at 37° C in an oven. The dried cellulose pellet was weighed, and percent content was calculated.

### Auxin metabolite profiling

Auxin metabolite profiling was performed as previously described (Novák et al. 2012). Five-day-old *gaut10-3* and Col-0 roots grown on 0.5× MS + 0.5% (15 mm) sucrose were harvested in pools of 25 mg and flash frozen; 5 biological replicates were analyzed for each genotype. Extraction and LC–MS were subsequently performed as published. Statistical analysis was performed using a nonparametric Wilcoxon rank-sum test with a *P* ≤ 0.1 to compare mean auxin metabolite concentrations between Col-0 and *gaut10-3*.

### IAA and IBA treatment assays

Homozygous *gaut10-3* (*gaut10* −/−) and *tob1-3* (*tob1* −/−) mutant lines were crossed to produce F1 individuals, which were verified by PCR to be heterozygous for both alleles (*gaut10* +/−; *tob1* +/−). F1 plants were selfed to obtain an F2 population containing double homozygous mutant seedlings of the PCR-verified genotype: *gaut10 −/−* and *tob1* −/−. F2 individuals were selfed to obtain F3 seeds of the desired genotypes. Genotyping for the *GAUT10*, *TOB1*, *gaut10-3*, and *tob1-3* alleles was conducted using PCR primers (Supplemental Table S6). Col-0, *gaut10-3*, *tob1-3*, and *gaut10-3 tob1-3* seeds obtained from the F3 individuals were surface sterilized using 50% bleach for 10 min, followed by 5 sterile water washes. After 3 d of imbibition in sterile water and cold stratification in the dark at 4 °C, the seeds were plated on 0.5 MS + 15 mm sucrose medium. Seeds were scored for germination under a long-day photoperiod (16 h light, 8 h dark) at 23 °C. Three days postgermination, the seedlings were treated with 10 *μ*m IAA and 10 *μ*m IBA and both dissolved in 95% ethanol or an equivalent volume of 95% ethanol (“mock”) for 72 h by transferring the seedlings to square Petri dishes containing fresh 0.5× MS + 15 mm sucrose supplemented with IAA/IBA or mock solvent. After 72 h after transfer, the seedlings were imaged on a flatbed scanner (Epson V600). Measurements of primary root lengths were performed using ImageJ for 70 to 80 replicates per genotype/treatment. A 1-way ANOVA followed by Tukey’s post hoc analysis was performed to test the statistical significance of the root length variations observed between these 4 genotypes.

### Accession numbers

Raw RNA sequence read data from this article can be found at the NCBI BioProject database under accession PRJNA694693 and ID 694693. All accession numbers for Arabidopsis genes mentioned in this study are listed in Supplemental Table S3.

## Supplementary Material

kiad465_Supplementary_Data

## Data Availability

The transcriptome data that support the findings of this study are openly available at the NCBI BioProject database under accession PRJNA694693 and ID 694693. The phenotyping, glycome, and auxin metabolite data are available within the supplementary material. All seed stocks are available upon request.

## Supplemental data

The following materials are available in the online version of this article.

**Supplemental Figure S1.** Transcript abundance of GAUT10 in different cell types of *A. thaliana* root meristem.

**Supplemental Figure S2.** Transcriptomic analysis of 5-d-old *gaut10-3* and Col-0 roots across 3 biological replicates.

**Supplemental Figure S3.** Additional auxin metabolite measurements.

**Supplemental Table S1.** Root phenotyping data of 5-d-old *gaut10-3* and Col-0 roots.

**Supplemental Table S2.** Glycome and monosaccharide composition data of 5-d-old *gaut10-3* and Col-0 roots.

**Supplemental Table S3.** Transcriptomic analysis data from 5-d-old *gaut10-3* and Col-0 roots with gene expressions averaged across 3 biological replicates.

**Supplemental Table S4.** *DR5:GFP* fluorescence quantification data.

**Supplemental Table S5.** Auxin metabolite concentrations of 5-d-old *gaut10-3* and Col-0 roots.

**Supplemental Table S6.** Primers used in this study.

## Dive Curated Terms

The following phenotypic, genotypic, and functional terms are of significance to the work described in this paper:

GAUT1 Gramene: AT3G61130
GAUT1 Araport: AT3G61130

GAUT11 Gramene: AT1G18580
GAUT11 Araport: AT1G18580

GAUT10 Gramene: AT2G20810
GAUT10 Araport: AT2G20810

NIT1 Gramene: AT3G44310
NIT1 Araport: AT3G44310

MYB34 Gramene: AT5G60890
MYB34 Araport: AT5G60890

GAUT15 Gramene: AT3G58790
GAUT15 Araport: AT3G58790

PEX11C Gramene: AT1G01820
PEX11C Araport: AT1G01820

GAUT14 Gramene: AT5G15470
GAUT14 Araport: AT5G15470

mur1 Gramene: AT3G51160
mur1 Araport: AT3G51160

HXK1 Gramene: AT4G29130
HXK1 Araport: AT4G29130

GAUT13 Gramene: AT3G01040
GAUT13 Araport: AT3G01040

MUR3 Gramene: AT2G20370
MUR3 Araport: AT2G20370
