# Supplementary material for: Changes in cell wall composition due to a pectin biosynthesis enzyme GAUT10 impact root growth
Source: Plant Physiol. 2023 Aug 22;193(4):2480–97. doi: 10.1093/plphys/kiad465 (PMC10663140; doi:10.1093/plphys/kiad465)
Supplement: kiad465_Supplementary_Data [file kiad465_supplementary_data.zip › Supplemental Data.pdf]

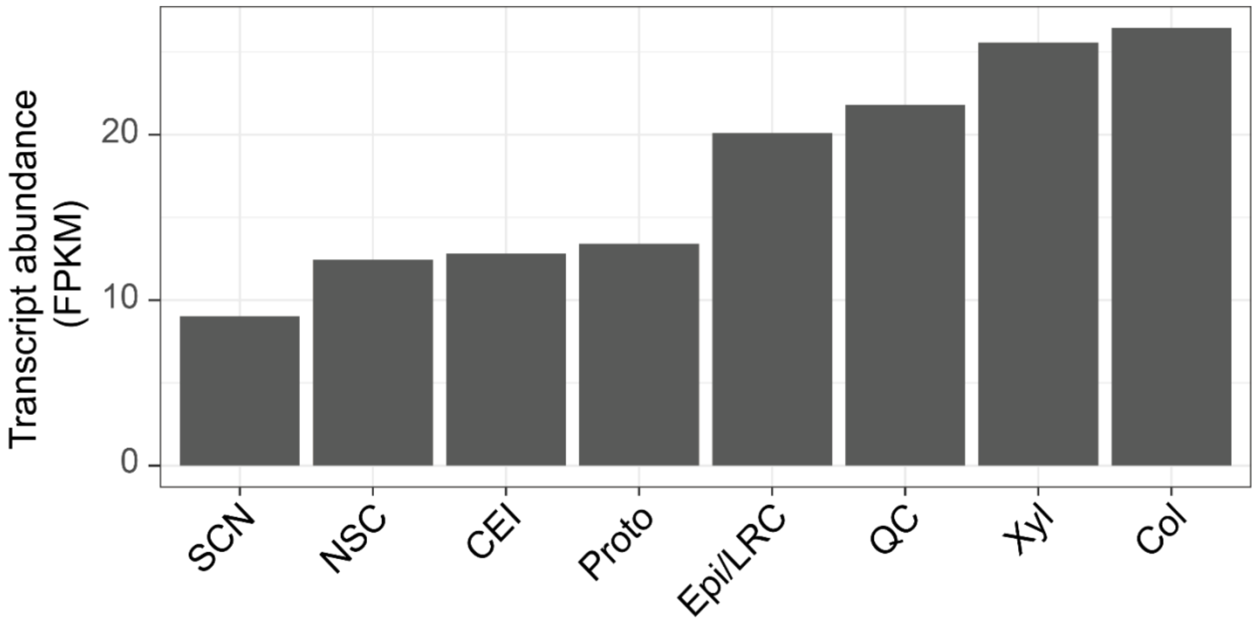

Supplemental Figure S1. Transcript abundance of GAUT10 in different cell types of *Arabidopsis thaliana* root meristem. *GAUT10* transcript abundance in Arabidopsis root stem cell populations from Clark et. al., 2019. Abbreviations used: SCN = stem cell niche, NSC = non-stem cells, CEI = Cortex/ endodermal initial stem cells, Proto = protophloem, Epi/LRC = epidermis/lateral root cap initials, QC = quiescent center, Xly = xylem initials, and Col = columella stem cells.

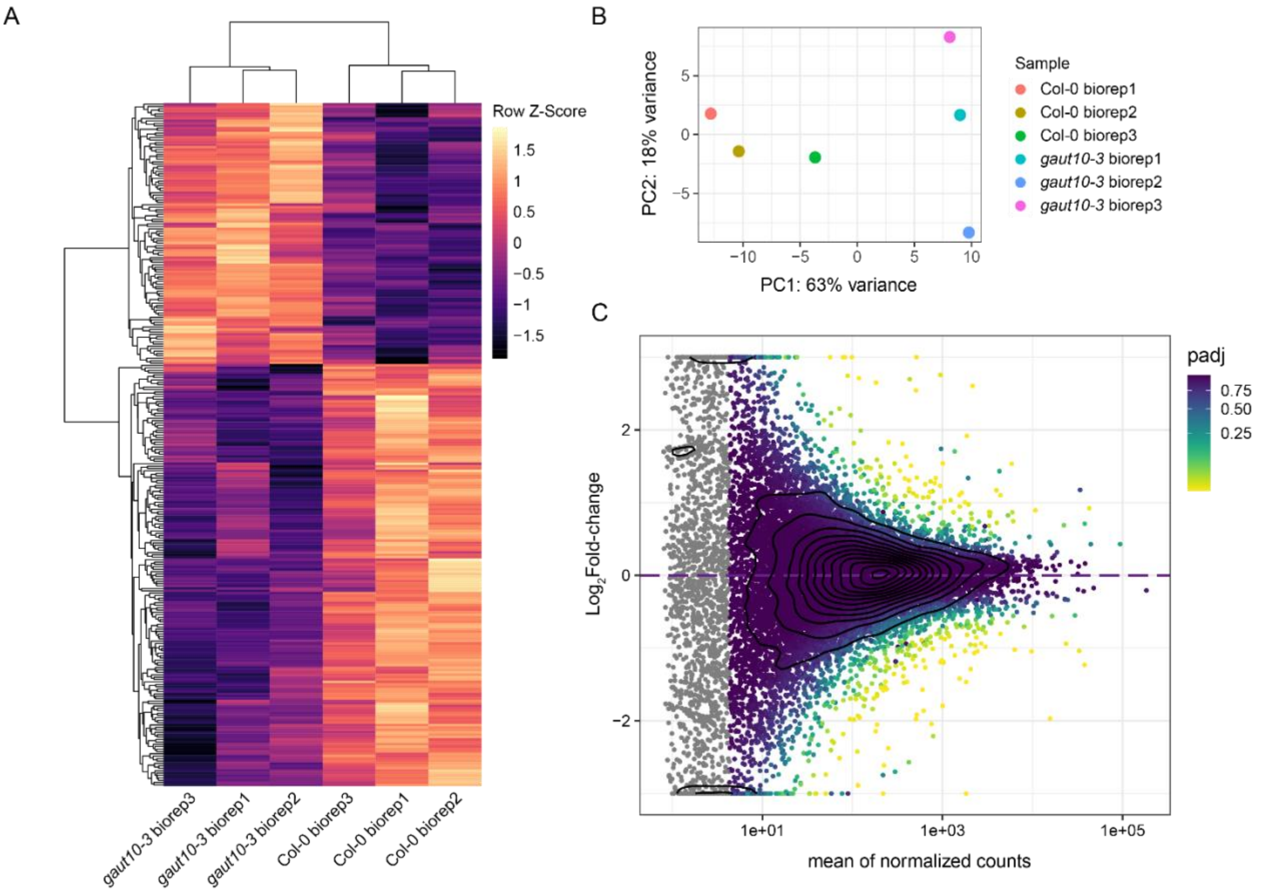

Supplemental Figure S2. Transcriptomic analysis of 5-day-old *gaut10-3* and Col-0 roots across three biological replicates. (A) Heat map shows the relative transcript abundance of all 285 differentially expressed genes clustered based on their row normalized Z-scores and biological replicates. (B) Principal component analysis (PCA) from DESeq2 package shows clustering of three biological replicates across two genotypes in a plane with PC1 as x-axis and PC2 as y-axis. (C) Dispersion plot from DESeq2 analysis showing mean normalized read counts in x-axis and Log_2FoldChange in y-axis, with genes color coded with their adjusted *P* values, where lighter shaded dots represent statistically significant DEGs.

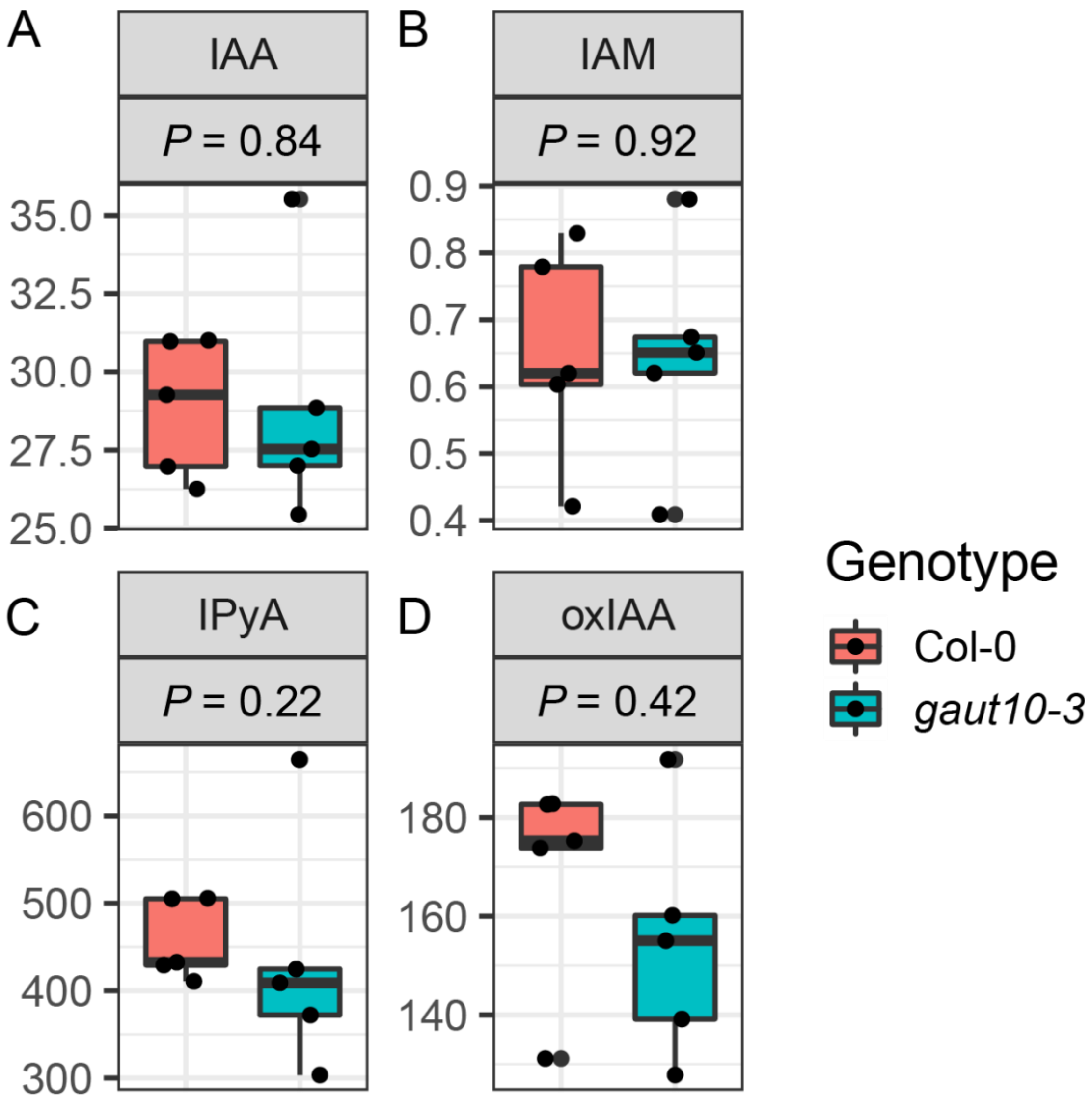

Supplemental Figure S3. Additional auxin metabolite measurements. Box and whisker plots show differences in concentrations (in picomoles per gram of fresh weight) of auxin metabolites between five-day-old *gaut10-3* and Col-0 roots averaged across 5 biological replicates. For all metabolites quantified, two sample nonparametric Wilcoxon rank sum tests followed by Benjamini-Hochberg correction for multiple testing were done to identify significantly altered metabolites with $P \leq 0.1$. (A) Indole-3-acetic acid (IAA) levels in Col-0 and *gaut10-3* (B) indole-3-acetaldoxime acid (IAM) levels in Col-0 and *gaut10-3* (C) indole-3-pyruvic acid (IPyA) levels in Col-0 and *gaut10-3* (D) 2-oxindole-3-acetic acid (oxIAA) levels in Col-0 and *gaut10-3*. Boxplots represent the five number summaries, where the center line is the median, box limits are upper and lower quartiles, whiskers are 1.5x interquartile range, and points are outliers.
